# Anti-Influenza Strategies Based on Nanoparticle Applications

**DOI:** 10.3390/pathogens9121020

**Published:** 2020-12-03

**Authors:** Klaudia Wieczorek, Barbara Szutkowska, Elzbieta Kierzek

**Affiliations:** 1 Institute of Bioorganic Chemistry, Polish Academy of Sciences, 61-704 Poznan, Poland; kwieczorek@ibch.poznan.pl (K.W.); bszutkowska@ibch.poznan.pl (B.S.); 2 NanoBioMedical Centre, Adam Mickiewicz University, 61-704 Poznan, Poland

**Keywords:** influenza, influenza virus, influenza vaccine, nanoparticles, nanotechnology, resistance, inhibition, delivery, antisense strategies

## Abstract

Influenza virus has the potential for being one of the deadliest viruses, as we know from the pandemic’s history. The influenza virus, with a constantly mutating genome, is becoming resistant to existing antiviral drugs and vaccines. For that reason, there is an urgent need for developing new therapeutics and therapies. Despite the fact that a new generation of universal vaccines or anti-influenza drugs are being developed, the perfect remedy has still not been found. In this review, various strategies for using nanoparticles (NPs) to defeat influenza virus infections are presented. Several categories of NP applications are highlighted: NPs as immuno-inducing vaccines, NPs used in gene silencing approaches, bare NPs influencing influenza virus life cycle and the use of NPs for drug delivery. This rapidly growing field of anti-influenza methods based on nanotechnology is very promising. Although profound research must be conducted to fully understand and control the potential side effects of the new generation of antivirals, the presented and discussed studies show that nanotechnology methods can effectively induce the immune responses or inhibit influenza virus activity both in vitro and in vivo. Moreover, with its variety of modification possibilities, nanotechnology has great potential for applications and may be helpful not only in anti-influenza but also in the general antiviral approaches.

## 1. Introduction

Influenza virus is a member of the *Orthomyxovirade* family of viruses [1]. Four influenza viruses can be distinguished: A, B, C (known for being human-infectious) and D (so far unconfirmed for being human-threatening) [2]. Among these, influenza A virus (IAV) and influenza B virus (IBV) have the potential to cause annual epidemics, known also as a seasonal flu. Moreover, IAV has a zoonotic potential, which means it can be easily transmitted from animals into humans. As a consequence, it has strong potential to evolve into a deadly pandemic strain and therefore is considered as one of the most human-threatening viruses [3].

**Figure 1:**
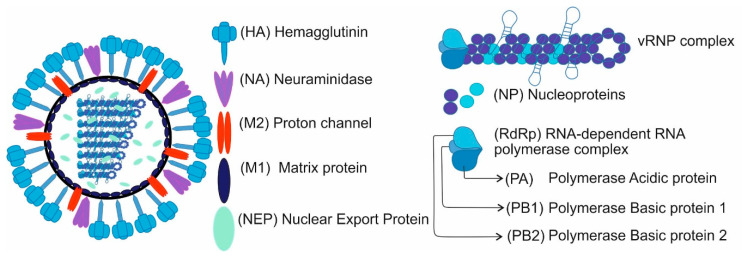
Scheme of influenza A virion structure. The virion surface is encrusted with the membrane proteins (hemagglutinin and nucleoprotein) and M2 proton channel proteins. The inner side of the virion is overlaid with M1 matrix protein. The 8 vRNP complexes and multiple copies of nuclear export protein are located in the virion interior.

The IAV genome consists of eight single-stranded, negative-sense RNAs ((-)RNA or vRNA) segments. RNAs along with viral proteins form eight viral ribonucleoprotein (vRNP) complexes. Each vRNP consists of the viral RNA segment bound to viral heterotrimeric RNA-dependent RNA polymerase (RdRp) and coated with nucleoproteins (NP). The influenza virus’ life cycle is entirely dependent on both RNA and viral polymerase. vRNA is a template for messenger RNA (mRNA) and complementary RNA (cRNA). The cRNA serves as a template for vRNA replication. The eight segments encode at least 16 proteins, including through alternative splicing and translation initiation [4]. These proteins include nucleoprotein, hemagglutinin (HA), neuraminidase (NA), matrix protein 1 and 2 (M1, M2), nuclear export protein (NEP), subunits of RNA-dependent RNA polymerase complex (PB1, PB2 and PA), as well as two non-structural proteins: PB1-F2 and NS1 (Figure 1).

The IAV genome undergoes two phenomena, antigenic shift and drift, that lead to the occurrence of new strains [5,6,7,8]. Different strains of the IAVs circulate annually as seasonal flus, and some of them can evolve into epidemic or pandemic strains [9]. Pandemics, written on the pages of modern history, showed that the influenza virus can be one of the most deadliest viruses [10]. The current SARS-CoV-2 pandemics is taking its toll around the world [11,12,13], recalling how deadly and severe for human health and devastating for the economy and daily life RNA viruses are. Moreover, many co-infections of influenza virus and SARS-CoV-2 have been observed worldwide and it seems that co-infections tends to strengthen the negative symptoms of the infection [14,15,16,17]. This broadens the already dangerous effects of influenza spreading and illness.

Vaccination is considered to be the most effective influenza prevention strategy [17]. The composition of the influenza vaccines is carefully estimated and published by the WHO every year, based on previous circulating influenza strains [18]. The vaccination’s effectiveness, monitored and published by the CDC, has ranged over the years from only 10% (season 2004–2005) to merely 60% (2010–2011) [19]. It is worth noting that the effectiveness varies between the particular subtypes, and in the previous 2018/19 season, it was significantly higher in H3N2 strains, as reported by Kissling et al. [20,21]. Although new generations of the universal vaccines have been developed, the perfect one has still not been found [22]. 

On the other hand, the constantly evolving genome of the influenza virus makes it resistant to existing antivirals. For that reason, there is an urgent need for developing new therapeutics and therapies. Most anti-influenza drugs target the viral proteins, so their effectiveness could be changed along with future viral mutations and might be only strain-specific effective [23,24]. Taking this together, the pandemic potential of the influenza virus should be studied even more closely. Many studies have applied different targets in the influenza virus, such as viral RNA or proteins, disrupting different viral processes including viral entry, replication or splicing as well as influencing cellular immune responses [25,26,27,28]. Developing new prevention methods and an effective treatment against the virus, based on up-to-date knowledge, is crucial. 

Nanotechnology has been with us for a very long time, although its use in science and medicine has grown rapidly over the last few years [29,30]. According to International Union of Pure and Applied Chemistry’s (IUPAC) definition recommendations, nanoparticles (NPs) are particles of any shape with approximately one of the dimensions in the 1 × 10^−9^ to 1 × 10^−7^ m range [31]. Usage of the NPs in life sciences gave life to new fields of science—nanomedicine and nanobiology [32]. These scientific fields complement each other and have opened up a broad range of applications in modern medicine—from diagnostic, molecular therapies, drugs, and their delivery to imaging biological structures on a molecular scale [33,34,35,36,37]. The possibility of choosing any size, morphology, porosity, and the possibility of any surface functionalization make NPs very attractive. It has been reported that the choice of particular NPs’ parameters is crucial while targeting specific cells or tissues is a major determinant to mitigate cytotoxic effects [38,39]. 

As the nanotechnology field grows, its applications in anti-influenza therapies and prevention methods are expanding. Intensive research on nanotechnology-based antivirals and their influence on the viral life cycle and cell itself broadens the knowledge concerning virus biology, and contributes to the development of nanomedicines. This review focuses on nanotechnology trends towards the facilitation of existing anti-influenza strategies and new methodologies applying NPs against influenza viruses.

## 2. Influenza Virus Inhibition Strategies Based on the Use of Nanoparticles

The nanosize and numerous possibilities of NPs’ functionalization make them excellent carriers of various antiviral agents. Here we present different strategies of using NPs to fight influenza virus infection: NPs as immunity-inducing vaccines, NPs used in gene silencing approaches, the use of NPs for drug delivery, and bare NPs which also exhibit anti-influenza properties (Table 1).

### 2.1. Nanotechnology in Vaccination Strategies

**Figure 2:**
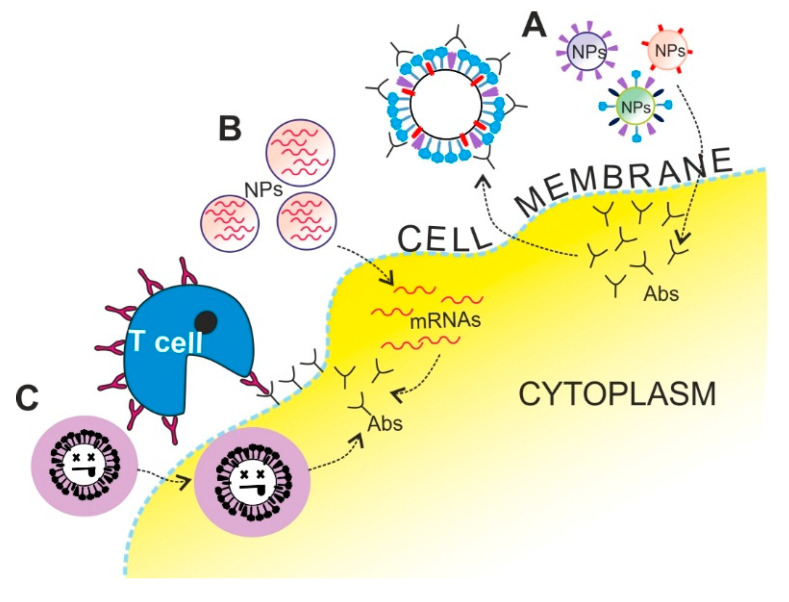
Schematic representation of different nanotechnology applications in vaccination strategies. The main goals are the stimulation of antibody (Ab) production and lymphocyte (T cell) activation. For this purpose, different vaccination strategies were used: viral antigens immobilized on nanoparticles’ (NPs) surfaces (A); viral self-amplifying mRNA encapsulated in NPs, which replicate inside cells, leading to antigen expression (B); or inactivated virus encapsulated in NPs (C).

The IAV is highly variable due to genetic drift and shift, and therefore the organism does not gain life-long immunity against the virus [5]. For that reason, new vaccine formulation must be developed and manufactured annually, which is a labor-extensive, time-consuming, and expensive process [69,70]. The WHO’s recommendation for northern and southern hemisphere influenza vaccine composition is predicted and introduced every year independently [71,72]. However, the vaccine’s effectiveness is variable between seasons and depends on prediction accuracy [73]. Hence, it is important to create new and faster methods for vaccine production to increase vaccination efficiency. Vaccination strategies rely on the delivery of the viral antigens immobilized on NPs (Figure 2 (A)), mRNA fragments coding the antigens (Figure 2 (B)) or inactivated virus (Figure 2 (C)) encapsulated in NPs into the cells as it is taking place during the viral infection (Table 2).

#### 2.1.1. Viral Antigen Vaccination Strategy Based on NPs Delivery

Commercial vaccines usually contain inactivated subunits or live attenuated influenza to induce immunity and produce antibodies (Abs) against HA and NA. However, vaccine production is a long process and antigenic drift can lead to a decrease in the vaccination’s effectiveness. Hence, it is very important to create new, faster to produce, and more effective vaccination methods.

When the viral infection occurs for the first time, the immune system is stimulated to produce specific Abs to gain immunity for the next viral encounter. Two types of Abs are produced during the infection: neutralizing Abs directly inhibiting the virion cell entry by interacting near the receptor binding site (RBS) of HA and non-neutralizing Abs that act indirectly by supporting other mechanisms such as phagocytosis, activating, complementing, and promoting antibody-dependent cellular cytotoxicity (Figure 2) [74]. Anti-influenza Abs can be further divided into Abs against external and internal proteins. There are three types of anti-external protein Abs: anti-HA, anti-NA and anti-M2. Moreover, organism can produce Abs against internal proteins: anti-nucleoprotein, anti-M1 and anti-PB1-F2 [74]. The viral antigen strategy relies on delivery of the viral antigens into the cells to produce anti-external Abs—anti-NA, anti-HA and anti-M2—as takes place during the viral infection.

##### Hemagglutinin Protein as a Viral Antigen in Vaccine Based on Nanoparticles

Hemagglutinin is a viral surface glycoprotein engaged in initiating the infection by binding the α-sialic acid residues presented on the hosts’ cell membrane [75]. It is a major determinant of pathogenicity and the main target of neutralizing Abs. Binding the HA to the sialic acid presented on the host’s cell surface is followed by the fusion of the viral envelope with the endosomal membrane and viral entry, allowing delivery of the virus to the cytoplasm [69,76]. 

Wang et al. designed dual-linker Au NPs conjugated with both recombinant trimetric HA from influenza A/Aichi/2/68 (H3N2) and flagellin (FliC) as an adjuvant [40]. FliC is a bacterial flagella protein, which can prompt the host’s innate signaling pathways through recognition by a TLR5-expressed antigen. The results of the in vivo study performed in mice show that levels of antigen-specific Abs are significantly increased after the nanovaccination. In the in vitro study, the Au NPs-HA/FliC conjugate was capable to trigger the TLR5 signaling pathways. As a result, antigenic epitopes were effectively processed and cross-presented by the antigen-presenting cells to induce the T cell-mediated immunity. In continuation of this study, Wang et al. investigated co-delivery of separately functionalized Au NPs-FliC and Au NPs-HA in an in vivo mice model. Although the IgG Abs levels were similar to the previous strategy, co-delivery stimulated CD8^+^ T cell response more efficiently [41].

Knuschke et al. synthesized biodegradable calcium phosphate (CaP) NPs encapsulating immunoactive TLR9 ligand (CpG) functionalized with the HA. Nanoconjugates were taken up by the dendritic cells in vivo and resulting in a strong T cell-mediated immune responses in immunized mice. Moreover, both methods of immunization (intraperitoneal and intranasal injections) were effective [42,43]. 

In a similar study, chitosan NPs were ionic cross-linked with sodium tripolyphosphate (CS/TPP) to deliver encapsulated HA antigen. The nanovaccine, tested in the mouse model, showed that HA encapsulated in CS/TPP could induce high numbers of IFN-γ-secreting cells in spleens while the naked HA vaccine could not. HA encapsulated in CS/TPP reduced the influenza morbidity and led to 100% protection against a lethal influenza virus dose in infected mice [44].

Kanekiyo et al. fused HA protein to ferritin, which naturally forms NPs composed of 24 identical units, leading to the assembly of the HA-generated eight trimeric HA spikes on its surface. Immunization of mice and ferrets elicited the reduction in HA Abs titers and induced neutralizing Abs against the HA structures: the stem and the RBS situated on the head. Nanovaccination protected against a broad spectrum of H1N1 IAV [45]. In a subsequent study, Kenekiyo et al. focused on the more conserved HA stem domain. Using HA-NPs elicited broadly cross-reactive Abs that fully protected mice and also partially protected ferrets against heterosubtypic H5N1 IAV [46].

The encapsulation method was also used to create recombinant H5 hemagglutinin trimer (H5_3_) trapped into polyanhydride NPs. The H5_3_ antigen works as a strong immunogen which induces high neutralizing Abs titers and enhances CD4^+^ T cell immune responses after the mice immunization. Moreover, H5_3_-polyanhydride NPs induce higher CD4^+^ T cell memory than H5_3_ antigen alone. However, the best results were obtained when H5_3_-polyanhydride NPs were used along with immunostimmulant (poly I:C). Such combination enhanced proliferative T cell responses in mice and inducted of Abs titers at earlier time [47]. In the continuation of this study, Ross et al. examined encapsulated H5_3_ in combined polyanhydride NPs and pentablock copolymer-based hydrogel to induce immunity against H5N1 IAV. Immunization in mice has induced high neutralizing Abs titers that were maintained for at least 70 days. While receiving the combination of nanovaccines, the mice had lower weight loss and reduced viral loads in the lung than mice vaccinated with each platform separately [48].

The subsequent research examined the immunogenicity stimulation with nanovaccine consisting of polyanhydride NPs encapsulating HA (H5M) antigen. The study confirmed the immunogenic potential of the nanovaccine, as level of immune responses against the H5N1 and H5N2 strains was significantly increased [49]. 

##### External Domain of the Matrix Protein 2 as a Viral Antigen in Vaccine Based on Nanoparticles

Antigenic drift and shift are mechanisms providing mutational potential of the influenza viruses [5]. In the antigenic drift, new types of HA and NA glycoproteins can evolve, while shifting can lead a new subtype to arise. For that reason, using a different viral antigen such as M2 protein might be very promising. The M2 is an integral membrane protein presented on the surface of nucleocapsid and the surface of virus-infected cells. It is engaged in processes such as cell entry and virion maturation [77,78]. The M2 protein is classified as a class I viroporin and consists of three domains: the extracellular N-terminal, internal hydrophobic, and C-terminal cytoplasmic domain [78]. The detailed study of the M2 protein showed that the Abs bind to the extracellular N-terminal region of the M2 protein (M2e) [79]. The natural immunity against the M2 protein in response to infection or induced by the conventional influenza vaccines is very weak [80,81]. However, combining the M2e domain with a protein carrier makes it highly immunogenic [82]. Therefore, using the M2e external domain of the M2 as a vaccine antigen aims to induce an adaptive immune response, which cooperates with the humoral anti-HA and anti-NA immunity. Moreover, the M2e antigen vaccine could overcome the shift and drift mechanisms and confront the virus with new immune responses. For that reason, the M2e is considered a universal vaccine candidate against the IAV [83]. 

In 2014, Tao et al. used a nanovaccine combining the highly conserved region of M2 protein attached to gold NPs (Au NPs). Mice were immunized intranasally with or without the addition of an adjuvant: soluble synthetic oligodeoxynucleotide containing unmethylated cytosine-guanine rich oligonucleotide motifs (CpG-ODN). The CpG-ODN has been used as a mucosal adjuvant to stimulate Th1-biased cytokines and a high response of IgG2a Abs level. The study confirmed that M2e-Au NPs conjugates with the addition of CpG-ODN enabled stimulation of M2e-specific IgG Abs, which could recognize both M2e and native M2 resulting in full protection of mice against the lethal dose of A/PR/8/1934 IAV [50]. The subsequent study showed that the molar ratio of M2e covering the Au NPs surface completely has significant effects on immune responses and overall protection against the lethal influenza dose. The group of mice vaccinated with the highest amount of free M2e exhibited 100% protection against influenza A/PR/8/1934 (H1N1) virus and showed the least weight loss [51]. The same mice vaccination schedule showed that the A/California/04/2009 (H1N1), A/Victoria/3/1975 (H3N2) and A/Vietnam/1203/2004 (H5N1) influenza virus strains led to 100%, 92%, and 100% protection, respectively [52].

M2e proteins also have been used to create nanosized virus-like particles (VLP). For this purpose, one to five copies of M2e were fused to the recombinant nodavirus capsid proteins to form the VLP. Mice immunization developed Abs specifically against M2e and the Abs titer was proportional to the copy numbers of M2e displayed on the VLP [53]. Similar results have also been obtained by De Filette et al., where they fused up to three copies of M2e to the hepatitis B core antigen, which self-assembled into a VLP [54,55].

##### Mix of Influenza Membrane Proteins as Viral Antigens Vaccines Based on Nanoparticles 

The localization of the antigen seems to be important while inducing an immune response. Interesting comparison of conjugated and encapsulated antigens (HA and NA from A/California/7/2009 (H1N1)) with trimethylaminoethylmethacrylate chitosan (TMC-chitosan) NPs was published by Liu et al. The group evaluated the antigen-specific immune responses in mice after nanovaccine nasal immunization. They concluded that antigen conjunct on TMC-chitosan NPs was more efficient to increase anti-H1N1 IgG level than antigen conjunct on pure TMC solution. The antigens conjunct on the surfaces of NPs were better recognized by APC than the antigens encapsulated into NPs. Functionalized NPs have been shown to stimulate macrophages and spleen lymphocytes to produce interleukins and interferons [56].

Hu et al. placed HA, NA and M1 antigens derived from A/Taiwan/S02076/2013 (H7N9) strain on the surface of the VLPs. VLPs were generated by co-infecting insect cells with three respective recombinant baculoviruses. In the experiments performed on mice and chickens, VLP immunization showed elevated Abs levels against NA and M1 proteins and increased antigen-specific cytokine production compared to the VLP-free protein formulation [57]. 

Further research focused on the combination of the recombinant HA and NA antigens. In detail, nanoadjuvants such as polyanhydride NPs and pentablock copolymer micelles were tested to check antiviral activity in the infected mice. The study indicates that two nanoadjuvants induced Abs titers significantly, resulting in reduced viral load and 100% protection in young mice [58].

Another interesting approach used biodegradable polylactic-co-glycolic acid (PLGA) NPs entrapping four conserved IAV H1N1 peptides: HA, nucleoprotein, PA and chimeric construct that express M2e on the surface loop of norovirus P particle (M2e-PP). Pigs vaccinated intranasally with PLGA NPs-peptides did not demonstrate any fever or flu symptoms. Vaccination without adjuvant also induced the virus-specific T cell responses in the lungs and reduced the viral load in the airways of pigs [59].

#### 2.1.2. Inactivated Influenza Virus Strategy Based on NPs Delivery

Interesting research into the incorporation into chitosan NPs of the inactivated virus along with immunostimulants such as CpG ODN or Quillaja saponin was performed by Deghan et al. Rabbit immunostimulation with CpG ODN showed better results in the induction of the humoral and Th1 responses compared to immunostimulation with Quillaja saponin [60,61]. 

Dhakal et al. evaluated the effectiveness of a polyanhydride NP encapsulating the inactivated swine IAV in pigs. The nanovaccine induces virus-specific lymphocyte proliferation and increases the frequency of CD4^+^CD8αα^+^ T helper and CD8^+^ cytotoxic T cells. The animals have lower viral antigens in the lungs and six to eight-fold reduction in nasal shedding of IAV compared to control animals [62]. Moreover, the nanovaccine prevented influenza-related symptoms, including fever.

Morçöl et al. used calcium phosphate NPs (CaP NPs) carrying inactivated IAV and tested them in mice. The CaP NPs as adjuvants induced higher hemagglutination inhibition, virus neutralization, and IgG Abs titers compared to the non-adjuvanted vaccine [63].

Alkie et al. investigated different types of inactivated IAV encapsulated in PLGA in chickens. They tested PLGA NPs encapsulating IAV alone or co-encapsulating CpG ODN adjuvant. The PLGA NPs co-encapsulating IAV and CpG ODN were also surfaced modified with mannan or chitosan. The authors reported that all tested nanovaccines induced an immune responses [64].

#### 2.1.3. mRNA Vaccines Strategy Based on NPs Delivery

mRNA vaccines are based on imitating the naturally occurring defective interfering particles (DIPs). Viral genes encoding structural proteins are replaced by mRNA fragments coding the antigens and deriving them in the viral-like NPs to the cells [84]. Thus, the mRNAs are replicated inside the vaccinated hosts’ cells, leading to the antigens’ expression, but without generating the infectious viral particles. Because the DIPs can activate the immune system by stimulating pathogen receptors—the pattern recognition receptors—the DIP-like NPs also have the same facility [84,85]. These kinds of vaccines are considered very promising to fight against infectious diseases, although they have some limitations. In comparison to standard vaccination strategy, this technology overcomes the antivector immunity responses and is egg-independent. Although the technology generates overall lower immunogenicity, it requires high doses and is considered as less stable [86].

One of the first studies using mRNA vaccines, was liposome-based NPs entrapping the mRNA encoding the nucleoprotein of A/Northern Territory/60/1968 (H3N2). The research performed in mice confirmed induced cytotoxic T lymphocytes, which were identical to those of the natural viral infection [65]. A similar approach has been tested by Bahl et al., where lipid NPs contained mRNA encoding HA protein of A/Jiangxi-Donghu/346/2013 (H10N8) or A/Anhui/1/2013 (H7N9) IAV strain. Immune responses have been studied in mice, ferrets, and nonhuman primates and measured by hemagglutination inhibition and microneutralization assays. Moreover, initial data from the first-in-human trial seem to confirm a robust immune response [66]. Another study used the self-amplifying mRNA (SAM^®^ technology) in lipid nanoparticles (LNP) vectors expressing nucleoprotein and M1 IAV proteins. Nanovaccines tested in mice confirmed the stimulation of nucleoprotein, M1, and HA-specific T-cells and HA-specific Abs [67]. The very recent study allowed the authors to select the perfect candidate for the universal, multi-targeting anti-influenza vaccine construction. The multi-targeting mRNA-LNPs vaccine has been confirmed to induce broad immune responses in mice using single and low dosage [68].

### 2.2. Nanotechnology in Gene Silencing Strategies

**Figure 3:**
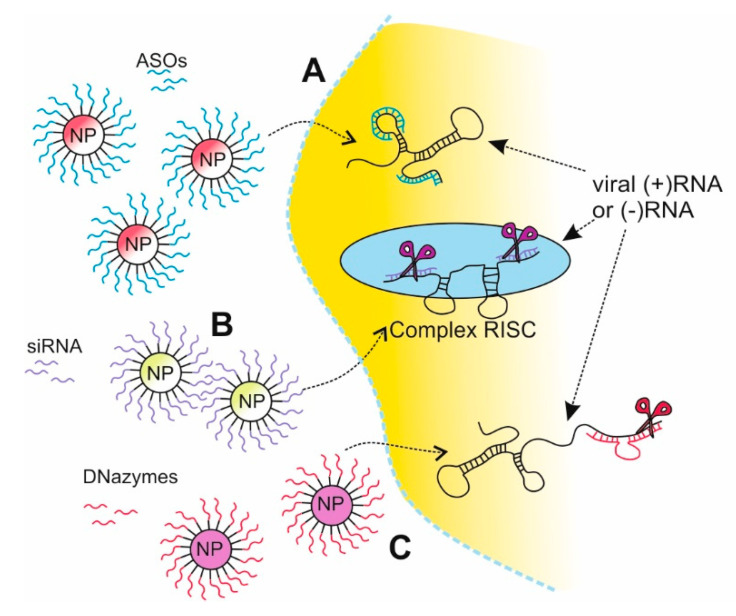
Schematic view of nanoparticle (NP) applications in gene silencing strategies. The NPs functionalized with oligonucleotides are used as a delivery system to the specific target locations in cells. Different oligonucleotide strategies are used to inhibit virus replication: blocking of accessible ssRNA regions with antisense oligonucleotides (ASOs) complementary to viral RNA (A); targeted degradation of viral mRNA with siRNAs (B); catalytic hydrolysis of viral RNA with DNAzymes designed to target viral RNA (C).

Gene silencing strategies are based on the natural mechanisms of gene inactivation. Silencers are synthetic nucleic acids able to repress gene expression by targeting particular fragments of the genome or transcriptome. The group of silencers includes antisense oligonucleotides (ASO), ribozymes, DNAzymes, short interfering RNAs (siRNA), microRNAs (miRNAs), short hairpin RNA (shRNAs), or peptide nucleic acids (PNAs). The evidence for their effectiveness in vivo has been provided in many, including those performed in our group [87,88,89,90,91,92]. However, the use of synthetic nucleic acids is limited due to imperfect systems of their introduction into cells. As for today, a few biochemical and physical transfection methods are available on the market [92,93]. However, they often influence the cellular condition, thus the search for new ones with reduced cytotoxicity and improved transfection efficiency is still in the spotlight [94]. In answer to the need, the NPs seem to be very promising as a tool for evaluation of new delivery systems. The multiple possibilities of the modification and functionalization, as well as the diversity of NPs types, making them very attractive potential transfection reagents (Figure 3).

#### 2.2.1. Antisense Oligonucleotides Strategy Based on NPs Delivery

The ASOs are single-stranded DNA oligonucleotides or RNA-modified oligonucleotides, generally 12–30 nt in length, which interact with the RNA of interest. There are several mechanisms of the ASO action: the activation of the RNase H cleavage of mRNA in DNA/RNA duplexes, creating a steric hindrance of the ribosome by binding ASO near the translational initiation site, destabilization and degradation of the transcript by ASO targeted near to the cap or the poly(A) of the mRNA, and destabilization or blocking the functional RNA structure (Figure 3 (A)). Other strategies include the inhibition of mRNA maturation and the induction of the mRNA precursor alternative splicing [95,96].

An interesting approach has been presented by Levina et al., who used ASO DNAs, targeting the noncoding 3′-terminal region of segment 5 (−)RNA. The ASOs were noncovalently immobilized on titanium dioxide NPs (TiO_2_ NPs) through the polylysine linker (PL). Nanocomposites used for the inhibition of H3N2 IAV replication in MDCK cells have shown a high antiviral activity simultaneously with the low cytotoxicity level. Nearly 50% of the virus inhibition concentration (IC50) was evaluated at 30 nM concentration for DNA (1.5 µg/mL of TiO_2_). Meanwhile, the 50% toxic concertation (TC50) was estimated to be 1800 µg/mL [97]. The subsequent study showed that the same ASO sequence covalently bound to PL and exhibited even higher antiviral activity than those associated via electrostatic interactions [98]. Next, they used the same nanocomposites to investigate the inhibition of the H1N1 and H5N1 strains. The antiviral activity of nanocomposites was very high (99,9%), probably due to targeting the conserved region of segment 5 IAV. The insertion of the mismatches has shown that the inhibition level is lower when the number of mismatches is higher [99]. In a subsequent study, using the ASOs targeting the most conservative regions of segment 5, both the negative (−)RNA and the positive (+)RNA sense strands of IAV were designed. Nanocomposites containing DNA targeting (−)RNA had higher antiviral activity than those targeting (+)RNA [100]. Moreover, Amirkhanov et al. studied the antiviral activity of TiO_2_-PL nanocomposites with DNA/peptide nucleic acids (PNA) heteroduplex. PNA targeting segment 5 of IAV RNA inhibited the IAV reproduction by 99.8% [101].

Levina et al. also presented ASO targeting the 3′-noncoding regions of viral (−)RNA of IAV segment 5 bound by electrostatic interactions to silicon–organic (Si-NH_2_) NPs. These NPs selectively and effectively inhibited the replication of the IAV in cell culture [102].

#### 2.2.2. siRNA Strategy Based on NPs Delivery

RNA interference (RNAi) is a post-transcriptional gene regulation process in which siRNA or miRNA, incorporated in the RNA-induced silencing complex (RISC), targets the mRNA in a sequence-specific manner (Figure 3 (B)). Interestingly, the RNAi is one of the naturally occurring antiviral mechanisms in mammalian cells, although its specificity is still not fully understood [106]. The RNAi mechanism has been used as an antiviral strategy against many viruses, including influenza [107]. Moreover, currently used methods for siRNA design, the simplicity of synthesis procedures, and the development of the new nanotechnology-based delivery methods make it an even more promising anti-influenza strategy. 

Frede et al. demonstrated multi-shell NPs consisting of a CaP core, covered with siRNAs encapsulated into PLGA (siRNA-loaded CaP/PLGA NPs) and coated with a final outer layer of polyethylenimine (PEI). They used siRNAs directly against the CCL-2, IP-10 and IFN-γ to reduce the gene expression in inflamed lung tissue. Mice treated with siRNA-loaded CaP/PLGA NPs revealed lower expression of targeted genes, resulting in reduced lung inflammation [103]. In the subsequent study, performed by Jamali et al., the chitosan NPs encapsulating siRNA targeting viral nucleoprotein were used. These siRNA-chitosan NP complexes inhibited IAV growth in cells and protected mice against a lethal viral dose [104].

#### 2.2.3. DNAzymes Strategy Based on NPs Delivery

DNAzymes, so far not widely explored, might be also attractive for nanoparticle-based antiviral application. DNAzymes offer another opportunity for NP application, so far not widely explored. DNAzymes are DNA fragments with catalytic capability to cleave complementary RNA (Figure 3 (C)). Although there are several artificial DNAzymes, so far natural ones have not been detected. Repkova et al. demonstrated the anti-influenza activity of DNAzymes immobilized on TiO_2_ NPs precovered with PL. The DNAzymes targeted the coding region of (+)RNA of IAV segment 5. The ability of TiO_2_ DNAzymes to cleave the target RNA has resulted in lower IAV replication by 2700-fold in MDCK cells [105].

### 2.3. Bare Nanoparticles Antiviral Strategy

Nanocarriers are not only a very useful tool for delivering drugs or nucleic acids, but bare NPs might be used as antivirals as well (Table 3). Nanotechnology is a very fast-growing field of science and has an infinite number of NP synthesis methods. As for today, the only limitation is an accurate idea of what effect is wished to be achieved. The nano-scale particles’ and materials’ main feature is that they exhibit different properties than at a greater scale. Moreover, different shapes, sizes and surface modifications of NPs are available, making them even more welcome for wider use in biomedical sciences [108].

Mehrbod et al. published the first research concerning the antiviral properties of the silver NPs (Ag NPs). The in vivo exposure of Ag NPs before and after the viral infection reduced the titer by 78.42% (at concentration 0.5 µg/mL) [109]. Next, the antiviral potential of Ag NPs against the H1N1 and H3N2 strains in both in vitro and in vivo was investigated by Xiang et al. The authors concluded that the best antiviral effect, which was nearly 98% of the survival rate in MDCK cells, was achieved after 72 and 96 h post-infection at 50 µg/mL Ag NP concentration [110]. The subsequent in vivo studies showed that 75% of mice treated with Ag NPs survived, while the IAV titer in the lungs was reduced by 2 log_10_ [111]. Different sizes of Ag NPs embedded into the chitosan matrix were tested as IAV inhibitors as well. Smaller Ag NPs were more effective than the larger one at similar Ag NP concentrations [112]. Fatima et al. synthesized Ag NPs (AgNO_3_) using *Cinnamomum cassia* extract and evaluated their activity against the H7N3 strain. A more significant inhibition level was observed when Ag NPs were incubated with the virus before infection, where the concentration of 200 µg/mL led to an inhibition level of 76% [113]. In a similar approach, the ultra-sonication method was used to synthesize Ag NPs with an aqueous extract from *Panax ginseng* roots. At concentrations of 0.02 and 0.25 M of Ag NPs, inhibition levels were obtained at very low levels: 7.10% and 15.12%, respectively, while the cytotoxicity was at 11.19% and 19.45%, respectively [114]. These results suggest that bare Ag NPs have stronger antiviral effects than NPs prepared by green synthesis. The reduced effectiveness of the NPs might be a result of the non-specific surface functionalization with naturally occurring compounds in the extract. Liang et al. reported that montmorillonite clay-based nano silicate platelets (NSP) surface-modified by anionic sodium dodecyl sulfate have antiviral properties against the IAV. Moreover, the NSP modified by Ag NPs did not suppress the infection of IAV at a concentration 10 µg/mL [115]. 

Kumar et al. studied the influence of size, shape, and surface properties of the pristine and polymer coated magnetite iron oxide (Fe_3_O_4_; IO) NPs. The cytoplasmic presence of IO NPs in the MDCK cells after 12 h has been confirmed by TEM. The pristine IO NPs did not reduce the IAV titer, although the polyethylene glycol (PEG) and polyvinyl pyrolidine (PVP) coated NPs showed 2.5 and 3.5 log of virus reduction with a concentration of 30 µg/mL [116]. Next, they tested glycine coated IO NPs against the H1N1 strain. They observed an 8-fold lower expression of the viral transcripts after the 24 h of viral infection, when 2 pg/mL concentration of IO NPs was used [117]. 

Ag NPs and IO NPs are not the only ones reported for their antiviral properties. Zinc oxide NPs (ZnO NPs) were also used against IAV in the post-exposure experiment. Bare and PEGylated ZnO NPs at the highest non-toxic concentration (75 µg/mL) reduced the viral titer to 1.2 and 2.8 log_10_ TCID50, respectively. Likewise, the cells exposed to ZnO NPs were tested before, during and after IAV exposure. The inhibitory effects were observed only when ZnO NPs were added 1 h after infection [118]. Sametband et al. tested anionic gold NPs (Au NPs) against several influenza A and B strains. The Au NPs were functionalized with mercaptosuccinic acid (Au NPs-MSA) or with mercaptoethanesulfonate (Au-MES NPs) molecules bound to the NPs’ surface through the thiol groups. Both particles inhibited influenza activity in the MDCK tissue culture; however, Au-MES NPs were more potent inhibitors in the lower concentrations. At the highest used Au-MES NPs concentration (0.5 mg/mL), the inhibition level was decreased by 97%. The virus-sulfonate interaction may play a unique role in blocking the virus activity [119]. Papp et al. designed Au NPs functionalized by sialic-acid-terminated glycerol dendron. They showed that sialic-acid linked to 14 nm Au NPs effectively bound to HA protein and inhibited viral replication. In that case, 30 min of preincubation of sialic-acid@Au NPs with IAV reduced the viral titer by 40%. Moreover, the cytotoxicity assay did not show any impact on cells’ viability [120]. TiO_2_ NPs in anatase phase also showed inhibition of the H9N2 strain. Moreover, the TiO_2_ NPs activated by UV light were shown to enhance the antiviral activity compared to controls [121]. In another study conducted by Thammakarn et al., calcium oxide NPs (CaO NPs) were prepared by grinding scallop shell powder into 500 nm NPs. The CaO NPs inactivated H7N1 IAV within 5 s of incubation [122]. The antiviral effects of zirconium dioxide (ZrO_2_) NPs were tested in H5N1-infected mice. The most effective NPs were selected from a variety of different sizes, surface charges, and compositions. The most successful NPs were 200 nm positively-charged ZrO_2_ NPs, which effectively protected mice against the IAV at a concentration of 100 mg/kg [123].

The exact mechanism of the antiviral effects of bare NPs is not fully understood. Xiang et al. suggested that Ag NPs may damage the viral envelope and thus inhibit the virus attachment to the host membrane. Ag NPs may also react with HA and block the bond formation between HA-cell membrane [111]. The mechanism of IO NPs antiviral activity in cells is still unidentified, although it has been shown that IO NPs in contact with IAV exhibit lipid peroxidase activities that catalyze viral lipid envelope peroxidation [124].

### 2.4. Nanotechnology in Delivery of Drugs Targeting Proteins

Some of the first anti-influenza drugs approved by the FDA were M2 ion channel inhibitors—amantadine and rimantadine (Table 4). A few decades later, it became clear that new resistant strains had evolved [125]. Today, they are classified as “historically used drugs” and are no longer approved to use by FDA [126]. Over the years, oseltamivir- and zanamivir-resistant (neuraminidase inhibitors, NAIs) strains have been described [127,128,129]. Strains resistant to NAIs are less frequent and for that reason oseltamivir and zanamivir are widely used, and new methods of their synthesis continue to be developed [130,131]. Another used NAI is peramivir, formerly region-restricted to China, Japan, South Korea, and the USA [25]. It is already authorized in European countries [132]. The last NAI, authorized in Japan only, is laninamivir [25,133]. Most recently, another drug, called baloxavir marboxil, was approved to use. The drug is an inhibitor of endonuclease activity of viral polymerase complex [134,135]. Considering the mutational nature of the IAV, known drugs might be insufficient in the not-so-distant future [24]. For that reason, development of the new anti-influenza strategies, in particular based on new generation technologies, is desirable.

**Figure 4:**
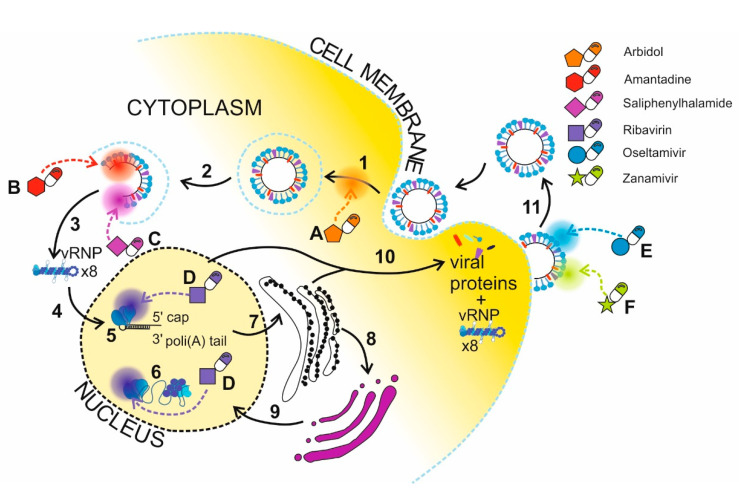
Schematic representation of anti-influenza drugs delivery via nanoparticles (A–F). Arbidol inhibits the formation of hemagglutinin-sialic acid bonding, and thereby blocks the viral entry (A). Amantadine blocks the viral M2 ion channel in order to disturb the uncoating process (B). Saliphenylhalamide blocks the acidification of endosomes and vRNP releasing (C). Ribavirin inhibits RdRp, thus disturbing vRNP formation and mRNA maturation processes (D). Oseltamivir and zanamivir are neuraminidase inhibitors, which blocks the release of the progeny virions (E and F, respectively). Influenza virus life cycle (1–11). Interaction between HA and sialic-acid followed by membrane fusion and viral entry to the cell (1). Endosome formation (2). Uncoating and vRNP releasing to the cytoplasm after pH changes catalyzed by M2 ion channel (3). vRNP transport to the nucleus (4). Genome replication and mRNA synthesis (5). mRNA maturation catalyzed by RdRp (6). Translation of viral proteins by ER and cytosolic ribosomes (7). Maturation of viral proteins at Golgi (8) followed by transport of the proteins to the nucleus for vRNP formation (9). Transport of vRNP and viral proteins to the cellular membrane for assembly and budding (10). Releasing of the progeny virions (11).

Li et al. and Lin et al. presented an interesting application of selenium NPs (Se NPs) to deliver anti-influenza drugs. Se NPs (70–200 nm) have been shown to be excellent carriers of oseltamivir, zanamivir, ribavirin, amantadine, and arbidol. These anti-influenza drugs successfully inhibited IAV replication [136,137,138,139,140]. The same authors tested Ag NPs as nanocarriers of amantadine, oseltamivir and zanamivir. The research showed effective inhibition of the IAV [141,142,143]. Figure 4 presents the influence of the particular drugs on the influenza life cycle.

NPs can also be used to deliver poorly soluble drugs. One of them is saliphenylhalamide (SaliPhe), which inhibits endosome acidification, an essential process for successful IAV infection (Figure 3 (C)). Bimbo et al. used thermally hydrocarbonized porous silicon NPs (THCPSi NPs) as a drug carrier to increase the solubility of SaliPhe. As a result, THCPSi@SaliPhe NPs inhibit the infection of IAV, and exhibit in vitro stability and low cytotoxicity [144].

## 3. Conclusions

The world of nanotechnology is rapidly expanding and showing new possibilities for the use of NPs in anti-influenza research and flu prevention. So far, we have been able to create NPs with a multitude of shapes, sizes, porosities, surface functionalizations, and other properties. Moreover, modifications of NPs can significantly alter their antiviral efficacy [108,112,116,123]. In this review, we presented various strategies for using NPs to fight influenza virus infection. NPs were used as advanced delivery vehicles for different antiviral agents, adding new functionality for known strategies. Additionally, NPs themselves appear to have anti-influenza properties. Besides the facilitation of potential drug development, the other category of nanotechnology application is usage in the delivery of immuno-inducing flu vaccines. Although the nanotechnology-based strategies and their antiviral application are very promising, the constantly mutating IAV genome is challenging and nanotechnology cannot overcome mutation problems. However, most of the strategies may be used for a wide range of IAV strains or can be easily adapted to new emerging strains. The NPs are excellent delivery agents of proteins, nucleic acids, anti-proteins drugs and vaccines. However, there is a need to ensure the safety of used NPs not only for human organisms but also for the environment in a long-term perspective. Possible cytotoxicity effects, drug–drug interactions or variable properties of each NP need to be carefully evaluated to take full benefit from the nanotechnology [145]. It is important to study the influence of particular NPs in in vivo systems. The good news is that there are already many NP-based antivirals that have shown minimal or no cytotoxic effects and might be considered as the therapeutics of the near future [118,146,147]. The safety of the nanotechnological products used in medicine is monitored and approved by the FDA [34,148]. There are already several cases of FDA-approved NP-based antivirals against different viruses, including influenza [145,149,150]. 

## Figures and Tables

**Table 1:** Nanotechnology-based anti-influenza vaccines.

Strategy	NPs Material/Size	Influenza Virus Strain (Subtype)	In Vitro/in Vivo Studies	Vaccine	Ref.
HA as an viral antigen	Au NPs/18 nm	A/Aichi/2/1968 (H3N2)	JAWS II, BMDC/mice	IN	[40]
		A/Aichi/2/1968 (H3N2)	HEK 293/mice	IN	[41]
	CaP NPs/250 nm	A/Puerto Rico/8/1934 (H1N1)	Splenocytes, dendritic cells, T cells/mice	SC, IN	[42]
		-	Splenocytes, dendritic cells, T cells/-	-	[43]
	chitosan NPs/300 nm	A/Brisbane/59/2007(H1N1)	-/mice	IN	[44]
	ferritin NPs/37 nm	A/New Caledonia/20/1999 (H1N1), A/Puerto Rico/8/1934 (H1N1), A/Singapore/6/1986 (H1N1), A/Brisbane/59/2007 (H1N1), A/California/04/2009 (H1N1), A/Beijing/262/1995 (H1N1), A/Solomon Islands/3/2006 (H1N1), A/Perth/16/2009 (H3N2), B/Florida/04/2006	-/mice, ferrets	SC	[45]
	ferritin NPs/no data	A/New Caledonia/20/1999 (H1N1), A/South Carolina/1/1918 (H1N1), A/California/04/2009 (H1N1), A/Singapore/6/1986 (H1N1), A/Canada/720/2005 (H2N2), A/Vietnam/1203/2004 (H5N1), A/Indonesia/5/2005 (H5N1), A/Anhui/1/2013 (H7N9), A/Hong Kong/1074/1999 (H9N2)	-/mice, ferrets	SC	[46]
	polyanhydride NPs/200 nm	A/Whooper Swan/Mongolia/244/2005 (H5N1), A/Vietnam/1203/2004 (H5N1)	-/mice	SC, IN	[47]
	polyanhydride NPs/200 nm	VNH5N1-PR8/CDC-RG (a reassortant virus strain containing the HA gene with a modified basic amino acid cleavage site) segment, the neuraminidase gene segment from the A/Vietnam/1203/2004 (H5N1) virus and the six internal gene segments of A/Puerto Rico/8/1934 (H1N1) virus generated by reverse genetics technology)	-/mice	SC	[48]
	polyanhydride NPs/200 nm	A/Vietnam/1203/2004 (H5N1), A/Indonesia/5/2005 (H5N1), A/Hong Kong/482/1997 (H5N1), A/Chicken/Qalubia-Egypt/1/2008 (H5N1), A/northern pintail/Alaska/622/2012 (H5N2), A/northern pintail/Alaska/622/2012 (H5N2), A/American green-winged teal/Alaska/472/2014 (H5N2), recombinant A/Puerto Rico/8/1934 (H1N1), recombinant A/chicken/Vietnam/NCVD5/2003 (H5N1)	-/chickens	SC	[49]
	Au NPs/12 nm	A/Puerto Rico/8/1934 (H1N1)	-/mice	IN	[50]
				IN	[51]
		A/California/04/2009 (H1N1), A/Victoria/3/1975 (H3N2), A/Vietnam/1203/2004 (H5N1)	MDCK/mice	IN	[52]
	VLPs/30 nm	no data	-/mice	SC	[53]
	VLPs/no data	mouse adapted recombinant X47virus (H3N2) (A/Victoria/3/1975 (H3N2) x A/PuertoRico/8/1934 (H1N1))	-/mice	IN	[54]
		mouse adapted recombinant X47virus(H3N2) (A/Victoria/3/1975 (H3N2) x A/PuertoRico/8/1934 (H1N1))	-/mice	SC	[55]
mix of virus membrane proteins as a viral antigen	chitosan NPs/140 nm	A/California/7/2009 (H1N1)	RAW 264.7/ mice	IN	[56]
	VLPs/114 nm	A/Taiwan/S02076/2013 (H7N9)	-/mice, chickens	SC	[57]
	polyanhydride NPs/200 nm	A/Puerto Rico/8/1934 (H1N1)	-/mice	SC	[58]
	PLGA NPs/260 nm	A/South Carolina/1/1918 (H1N1), A/New Caledonia/20/1999 (H1N1)	-/pigs	IN, IT	[59]
inactivated virus strategy	chitosan NPs/581 nm	A/New Caledonia/20/1999 (H1N1)	-	-	[60]
		A/New Caledonia/20/1999 (H1N1)	-/rabbits	SC, IN	[61]
	polyanhydride NPs/200 nm	A/Swine/Ohio/24366/2007 (H1N1), A/Swine/Ohio/FAH10-1/2010 (H1N2)	-/pigs	IN	[62]
	CaP NPs/450–500 nm	A/California/7/2009 (H1N1)	MDCK/mice	IM	[63]
	PLGA NPs/624 nm chitosan@PLGA NPs/819 nm mannan@PLGA NPs/719 nm	A/Duck/Czech/1956 (H4N6)	chicken	MC, SC	[64]
mRNA strategy	lipid NPs/ no data	A/Northern Territory/60/1968 (H3N2)	-/mice	IJ	[65]
	lipid NPs/80–100 nm	A/Anhui/1/2013 (H7N9), A/Jiangxi-Donghu/346/2013 (H10N8)	-/mice, ferrets, cynomolgus monkeys (cynos)	IJ	[66]
	lipid NPs/130–142 nm	A/Puerto Rico/8/1934 (H1N1), mouse-adapted A/HongKong/1/1968 (H3N2), A/California/7/2009 (H1N1)	BHK/mice	IM	[67]
	lipid NPs/~80 nm	Recombinant IAV with: the HA and NA from A/Singapore/GP1908/2015 (H1N1) and remaining proteins from A/Texas/1/1977 (H3N2), A/Michigan/45/2015 (H1N1), A/New Caledonia/20/1999 (H1N1) and A/Puerto Rico/8/1934 (H1N1). Recombinant chimeric IAV with: HA head domain from A/mallard/Sweden/81/2002 (H6N1), HA stalk domain from A/California/04/2009 (H1N1), NA from A/mallard/Sweden/86/2003 (H12N5) and remaining proteins from A/Puerto Rico/8/1934 (H1N1). Recombinant IAV with the polybasic cleavage site removed from the A/Vietnam/1203/2004 (H5N1), the N8 from A/mallard/Sweden/50/2002 (H3N8) and remaining proteins from A/Puerto Rico/8/1934 (H1N1). Recombinant IAV with: the HA and NA from A/chicken/Hong Kong/G9/1997 (H9N2) and remaining proteins from A/Puerto Rico/8/1934 (H1N1) and A/Hong Kong/4801/2014 (H3N2).	NIH-3T3, MDCK, HEK293T/ mice	IJ	[68]

BMDCs: bone marrow-derived dendritic cells; HEK: human embryonic kidney 293 cells; “-”: not tested; MDCK: Madin-Darby canine kidney cells; BHK: baby hamster kidney cells; ID: intradermal; IM: intramuscular; IJ: injections; IN: intranasal; IT: intratracheal; MC: mucosal; SC: subcutaneous.

**Table 2:** Anti-influenza gene silencing strategies based on nanotechnology.

Strategy	NPs Material/Size	Influenza Virus Strain (Subtype)	In Vitro/In Vivo Studies	Vaccine	Ref.
antisense oligonucleotides	TiO_2_ NPs/ 5 nm	A/Aichi/2/1968 (H3N2)	MDCK/-	-	[97]
		A/Aichi/2/1968 (H3N2)	MDCK/-	-	[98]
		A/Salekhard/01/2009 (H1N1), Aichi/2/68 (H3N2), A/chicken/Kurgan/05/2005(H5N1)	MDCK/-	-	[99]
		A/Salekhard/01/2009 (H1N1), Aichi/2/68 (H3N2), A/chicken/Kurgan/05/2005(H5N1)	MDCK/-	-	[100]
	TiO_2_ NPs/ 4–6 nm	Aichi/2/1968 (H3N2)	MDCK/-	-	[101]
	Si-NH_2_ NPs/ 1.2–1.5 nm	A/chicken/Kurgan/05/2005 (H5N1)	MDCK/-	-	[102]
siRNA	CaP NPs/ 145 nm	A/Puerto Rico/8/1934 (H1N1)	-/mice	IN	[103]
	chitosan NPs/278 nm	A/Puerto Rico/8/1934 (H1N1)	Vero/mice	IN	[104]
DNAzymes	TiO_2_ NPs/5 nm	A/chicken/Kurgan/05/2005 (H5N1)	MDCK, HeLa/-	-	[105]

MDCK: Madin-Darby canine kidney cells; “-”: not tested; IN: intranasal.

**Table 3:** Bare nanoparticles as new anti-influenza therapeutics.

Strategy	NPs Material/Size	Influenza Virus Strain (Subtype)	In Vitro/In Vivo Studies	Vaccine	Ref.
silver NPs	Ag NPs/no data	A/New Caledonia/20/1999 (H1N1)	MDCK/-	-	[109]
	Ag NPs/10 nm	A/Puerto Rico/8/1934 (H1N1)	MDCK/-	-	[110]
	Ag NPs/10 nm	A/Human/Hubei/3/2005 (H3N2)	MDCK/mice	IN	[111]
	Ag NPs/4–13 nm	A/Puerto Rico/8/1934 (H1N1)	MDCK/-	-	[112]
	Ag NPs/42 nm	no data (H7N3)	Vero/-	-	[113]
	Ag NPs/ 5–15 nm	A/Puerto Rico/8/1934 (H1N1)	MDCK/-	-	[114]
	silicate platelet@Ag NPs/ 80 × 80 × 1 nm	A/WSN/1933 (H1N1)	MDCK, A549/-	-	[115]
iron oxide NPs	Fe_3_O_4_ NPs/8–10 nm	A/2009 (H1N1)	MDCK/-	-	[116]
	Fe_3_O_4_ NPs/8–10 nm	A/Puerto Rico/8/1934 (H1N1)	MA104/-	-	[117]
zinc oxide NPs	ZnO NPs/16–50 nm	A/Puerto Rico/8/1934 (H1N1)	MDCK/-	-	[118]
gold NPs	Au NPs/5 nm	A/Israel/119/2009 (H1N1), A/Brisbane/10/2007 (H3N2), A/Puerto Rico/8/1934 (H1N1), B/Brisbane/60/2008, B/Shandong/7/1997	MDCK/-	-	[119]
	sialic-acid@Au NPs/2 and 14 nm	A/X-31 (H3N2)	MDCK, CHO/-	-	[120]
titanium dioxide NPs	TiO_2_ NPs/50 nm	no data (H9N2)	MDCK/-	-	[121]
calcium oxide NPs	CaO NPs/500 nm	A/duck/Aomori/395/2004 (H7N1)	MDCK/-	-	[122]
zirconium dioxide NPs	ZrO_2_ NPs/200 nm	A/chicken/Henan/1/2004 (H5N1)	-/mice	IN	[123]

MDCK: Madin-Darby canine kidney cells; “-”: not tested; CHO: chinese hamster ovary; IN: intranasal.

**Table 4:** Delivery of anti-influenza drugs by nanocarriers.

Drug	NPs Material/Size	Influenza Virus Strain (Subtype)	In Vitro/In Vivo Studies	Vaccine	Ref.
oseltamivir	Se NPs/ 70–200 nm	no data (H1N1)	MDCK/-	-	[136]
zanamivir		A/Hubei/74/2009 (H1N1)	MDCK/-	-	[137]
ribavirin		no data (H1N1)	MDCK/-	-	[138]
amantadine		A/Guangdong/1/2009 (H1N1)	MDCK/mice	IN	[139]
arbidol		no data (H1N1)	MDCK/mice	IN	[140]
amantadine	Ag NPs/ 2–3 nm	no data (H1N1)	MDCK/-	-	[141]
oseltamivir		no data (H1N1)	MDCK/-	-	[142]
zanamivir		A/Hubei/74/2009 (H1N1)	MDCK/-	-	[143]
saliphenylhalamide	THCPSi NPs/129 nm	A/WSN/1933 (H1N1), A/Puerto Rico/8/1934-NS116-GFP (H1N1)	MDCK, monkey Vero-E6/-	-	[144]

MDCK: Madin-Darby canine kidney cells; “-”: not tested; IN: intranasal.
